# How do policymakers involve citizens in advancing health? A mixed-method qualitative study in municipalities in the Netherlands

**DOI:** 10.3389/fpubh.2025.1708209

**Published:** 2025-11-24

**Authors:** Helene R. Voogdt-Pruis, Diederick E. Grobbee, Sanne A. E. Peters, Charisma Hehakaya, Marielle Jambroes, Kerstin Klipstein-Grobusch, Astrid Janssens

**Affiliations:** 1 Department of Global Public Health and Bioethics, Julius Center for Health Sciences and Primary Health, University Medical Center Utrecht, Utrecht University, Utrecht, Netherlands; 2 The George Institute for Global Health, School of Public Health, Imperial College London, London, United Kingdom

**Keywords:** public policy, health policy, prevention, health promotion, local government, stakeholder participation, citizen science, community engagement

## Abstract

**Introduction:**

Citizen involvement in health policymaking is becoming imperative. Yet, implementing this practice in local policymaking often proves challenging. This study explores how local policymakers in the Netherlands experience and approach citizen involvement in health promotion policymaking.

**Methods:**

In 2023, a stratified random sample of 65 out of 342 Dutch municipalities was selected to participate in this mixed-method study, combining survey responses and interviews. Policymakers from 22 Dutch municipalities participated. Descriptive quantitative analysis and content analysis was applied.

**Results:**

Policymakers widely value citizen involvement in health policymaking for understanding community perspectives and needs, identifying priorities and agenda setting, identifying solutions and building public trust, accountability and transparency. In day-to-day practice, citizens are mainly approached for prioritising policy issues or for brainstorming on solutions. A range of consultation methods is used, sometimes in collaboration with public health services or research institutions. Time commitment and ensuring data quality are the main challenges for meaningful citizen involvement next to organizational challenges. Concerns about the quality of the data relates to low response rate and selective response. At times, policymakers may not have the appropriate resources or expertise to facilitate effective citizen involvement. In the domain of preventive health, the following questions on citizen involvement arose: How to address needs, how to include population groups with greater health risks, how to understand citizens’ context well?

**Conclusion:**

Citizen involvement is a recognised and frequently applied practice in local health policymaking. However, efforts remain fragmented and face operational barriers. These challenges are particularly pronounced in the area of health promotion policymaking. Stronger support and local capacity can improve the inclusiveness and impact of citizen engagement, particularly in preventive health policy.

## 1 Introduction

Noncommunicable diseases are strongly influenced by preventable factors such as including poor dietary habits, physical inactivity, alcohol and tobacco consumption, and environmental conditions like air pollution and extreme heat. In response, governments worldwide are encouraged by international organisations, such as the World Health Organization and the World Heart Organisation, to prioritise population health and wellbeing through preventive strategies (1–4). At national, regional and local governmental level, policymakers tailor these preventive policies to specific community needs and contexts, often requiring collaboration across sectors – including with citizens (5–12). The successful implementation of preventive solutions can only be achieved through collaboration, given the complexity of challenges such as the distribution of benefits and costs across sectors or stakeholders, tensions with personal and cultural beliefs, and the delayed or intangible nature of preventive outcomes (3, 5).

Citizen involvement should be regarded as an integral component of health policymaking, encompassing a broad range of health-related issues — including health and care services, the living environment (such as transport, green spaces, local shops, and meeting places), and opportunities for social engagement (9, 12, 13). The involvement ranges from mere observation to the exercise of power. Citizen involvement is a complex concept and practice, with multiple purposes, meaning, levels and methods (5, 14–16), in which the term ‘citizen science’ is often restricted to the involvement of citizens in scientific data collection or analysis (17–19).

Citizen involvement in government decision making has gained attention as a way to improve government performance, decision legitimacy, citizen responsiveness, and trust in government (13, 14, 20). Yet, citizen involvement is not without challenges. A major challenge of citizen involvement is the representativeness of participating citizens and the validity of the results, as mostly higher educated and affluent citizens participate (10, 16, 19, 21). On the side of policymakers, main challenges reported are the availability of time and resources for citizen involvement and the difficulty to synthesize and integrate citizens’ input in the different stages of the policymaking process (10, 12, 22, 23).

In the Netherlands, national programmes on health promotion (7, 8) have recently placed stronger emphasis on citizen involvement in local policy making. While the literature reflects a wide spectrum of citizen involvement initiatives and frameworks worldwide and in the Netherlands, this study was initiated to investigate how citizen involvement is currently practiced by local Dutch policymakers. Our study draws on three complementary theoretical frameworks. First, participatory governance (24) Participatory Governance provides a lens to examine the institutional embedding and design of participatory processes—specifically, who is involved, how input is incorporated, and what impact participation has on policy outcomes. Second, Street-Level Bureaucracy (25) informs our understanding of how frontline policymakers interpret and adapt citizen involvement within the realities of their daily work. Third, we draw on ‘Normalisation Process Theory’ (26) to explore how new practices—such as citizen involvement - are integrated and sustained within routine health policy processes. This paper aims to address the gap of limited understanding on daily practice of citizen involvement in local health policymaking by exploring the motivations, methods and challenges for citizen involvement as experienced by Dutch policymakers. Using a mixed-method approach, we provide insights not only into current practices, but also into the structural and contextual factors that shape them — offering practical implications for strengthening citizen engagement in preventive health policy.

## 2 Materials and methods

### 2.1 Design of the study

In 2023, we conducted a study among municipal policymakers who are involved in local health promotion. Through questionnaires followed by semi-structured interviews, we investigated the experiences and perspectives of these municipal policymakers on citizen involvement in the local policymaking on health promotion and prevention. We used the COREQ checklist for reporting (27). The study was carried out among the 342 municipalities in the Netherlands^.^ Municipalities constitute the third-level administrative public bodies and function as subdivisions of 12 provinces (28). Municipalities possess a level of autonomy in policymaking and bear responsibility for a diverse range of public services (29)^.^ These services encompass land-use planning, public housing, management and maintenance of local roads, waste management and social security (30). Since 2015, the Dutch government delegated public health tasks to the municipalities. This allows municipalities to collaborate with a variety of stakeholders in the development of local prevention and health promotion policies. The municipalities vary in population density. Small municipalities have less than 50 thousand inhabitants (*n* = 250) and medium and large municipalities have 50–100 thousand (*n* = 60) and at least 100 thousands (*n* = 32) inhabitants. The 12 provinces cover 6 (Flevoland) to 56 (Noord-Brabant) municipalities (28). Each municipality is obligated to establish and maintain a local public health service (GGD). They cooperate with other municipalities to organize such services. In 2024, 25 regional public health services covered all municipalities. GGDs are managed by the councillors of participating municipalities (31).

### 2.2 Research team and reflexivity

Two members of the research team (DG and HVP) serve as chairs of one of the 25 thematic tracks of the Dutch Research Agenda – *Health Care Research, Disease Prevention, and Treatment* (32). This thematic track has identified a significant knowledge gap, particularly regarding the role of municipalities in promoting community health, for example: How can citizens be effectively involved in shaping health strategies? How can other actors contribute to prevention efforts? And how can cooperation between stakeholders be improved? It is important to note that citizens who are not in need of care or assistance may find it difficult to engage in prevention initiatives - especially when these seem irrelevant to their own lives or to the services municipalities offer. Another team member (AJ) holds a professorship in Public and Patient Involvement, offering in-depth expertise in the field of citizen participation. The municipalities and participants were approached by one researcher (HVP), a female researcher in user-centredness within integrated cardiovascular prevention, employed in a medical university centre in the Netherlands. All interviews with policymakers were held by this author, who did not have any prior relationship with the interviewees. This interviewer introduced the background of the study within the realm of the Dutch Research Agenda, to identify research and implementation gaps to be addressed in future research. Data analysis was performed by two researchers (HVP and CH). They both have a PhD degree within the health sector, without specific training or experience in public administration. The other members of the research team (SP, MJ, KKG) bring substantial expertise in public health practice, preventive strategies and engagement with different stakeholders and citizen groups.

### 2.3 Data collection

Between July and October 2023, a stratified random sample of 65 (19.0% of 342) municipalities was invited to participate in this study. The sample size of 65 municipalities was determined based on a balance between representativeness across regions and municipality sizes, and the available time and research capacity to conduct in-depth interviews and analyses. Stratified random sampling ensured diversity in rurality, population density, and geographic spread. Given this sampling strategy, we consider the selected municipalities to provide an in-depth reflection of the practical, day-to-day challenges that policymakers face in engaging citizens in local health policymaking (28). Invitations were sent by mail to the municipality department in charge of health promotion and prevention policy and, if applicable, citizen involvement. In case of non-response, a reminder mail was sent after two weeks. The invited municipality’s department selected a policymaker to participate in the study who met the inclusion criteria of the study, as shared by the researcher. Eligible participants were policymakers employed in the municipality focusing on supporting health and wellbeing and/or creating healthy environments, in roles involving policy or program planning. Work experience and job position were not limiting factors for eligibility. Policymakers were invited by email to participate in an online semi-structured interview, lasting a maximum of 30 min. These interviews focused on the experiences, views and plans regarding local health promotion policy and citizen involvement. Participants received a short questionnaire via mail as preparation for the online semi-structured interview. Participants were asked to answer according to the actual practice of the department. The questionnaire was adapted from a survey, consisting of 13 questions, used in a study to explore citizen involvement in chronic disease prevention among policy and practice stakeholders across Australia (15). The original Australian survey was informed by the literature on community engagement in public health research, policy, and practice (33). The Australian survey was translated into Dutch and adapted to the local policy context. The primary purpose of the questionnaire in our study was not to collect validated or generalisable quantitative data. Rather, it was used as a preparatory tool to help participants reflect on their local practices and challenges regarding citizen involvement. Participants were asked to describe the goals and activities of citizen involvement and to indicate the perceived benefits and challenges of their involvement concerning prevention and health promotion policy (Supplementary Appendix 1). The questionnaire required participants to complete an informed consent form before proceeding. Collected socio-demographic information of participants included age, gender, role, and areas of prevention activities. The questionnaire responses informed the topic guide for the semi-structured interviews, supporting participants to describe real-world practices.

The interviews had an average duration of 25 min (range 16–68 min) and were held without audience. Interviews were audio recorded and transcribed for analysis in Microsoft Word.

### 2.4 Data analysis

The questionnaire data were exported from Microsoft Word into Microsoft Excel and IBM SPSS V28 for descriptive statistics. The 5-point Likert scale responses were consolidated and the categories “1–2” (not at all – little) and “4–6” (somewhat – quite a lot – a great deal) were combined. While the questionnaire offered insight into the practice of citizen involvement in local health policymaking, the emphasis of the data analysis lay on the qualitative interview data in order to gain a deeper understanding of the considerations and challenges policymakers face in implementing it and what types of improvements are considered necessary by policymakers themselves. For the analysis of interview transcripts, we used directed content analysis (34). We primarily identified quotes that highlight the policymakers’ experiences and views on citizen involvement in health promotion policymaking. An coding scheme was developed deductively and refined through inductive coding as new insights emerged from the interviews. Differences were discussed and resolved through consensus, leading to adjustments in code definitions. The two researchers independently analysed part of the 22 questionnaires and transcripts. Subsequently, they discussed the most important results and quotes. To enhance the validity of our findings, we used methodological triangulation by integrating questionnaire data with qualitative interview insights. This integration occurred primarily at the analysis and reporting stages. Questionnaire responses were used to identify patterns, which were then explored in greater depth during the interviews. Bringing the two data sources together led to a deeper and more meaningful interpretation. For example, perceived barriers to citizen involvement reported in the questionnaire were illustrated and nuanced through in-depth reflections from interviewees. The results and key messages of the study were shared with all participating policymakers in the form of a concise PowerPoint presentation, accompanied by an invitation to provide comments, suggestions, or follow-up questions.

## 3 Results

### 3.1 Participants

A total of 22 municipal policymakers participated in the online semi-structured interviews and pre-sent questionnaire. Two policymakers participated either in the semi-structured interview or the pre-sent questionnaire. The participating municipalities were representative in terms of size: 72.7% small (<50,000 habitants), 22.7 medium (50.000–100.000 habitants) and 4.5% large (>100.000 habitants). No municipalities from two provinces (Friesland, Noord-Holland) did respond (Supplementary Appendix 2). The majority of the municipal policymakers were female, and the average age was 44.4 years (Table 1). Some policymakers were employed across several municipalities (18.8%, Table 1). The participating municipal policymakers worked predominantly on the domains of health, wellbeing and sports.

**Table 1:** Characteristics of Dutch policymakers who participated in the study, 2023.

Characteristics	Total
Municipalities	342
Invited municipalities	65 (100.0%)
Participating municipalities	22 (33.8%)
Policymakers who participated	
Female (portion, *n*)	68.2%, *n* = 22
Age (mean, range *n*)	44.6 years, 28–65, *n* = 21
Field of policy	All were into health, wellbeing, social support and/or sports
Job position	All were (senior) policy officer
Local versus regional active (portion, *n*)	81.3%, *n* = 16

### 3.2 Citizen involvement in policymaking on health promotion

In presenting the findings, we integrated quantitative patterns with qualitative insights to provide a comprehensive understanding of how citizen involvement is practiced and perceived. Although the descriptive statistics offer a sense of how commonly certain motives, challenges and approaches were mentioned by participating municipalities, they primarily serve to frame the qualitative findings.

### 3.3 Reasons for citizen involvement

Most municipalities consider citizen involvement in preventive health policymaking important (Table 2). Most selected primary objectives for citizen involvement were ‘understanding community perspectives and needs’, ‘identifying priorities and agenda setting for the community’, ‘identifying solutions’ and ‘building public trust, accountability and transparency’.

**Table 2:** Reasons for citizen involvement in local policymaking on health promotion as reported by policymakers from the municipalities involved in the study (*n* = 22), 2023.

Reasons for citizen involvement	Total
Priority of public engagement within the organization (*n* = 22) (% high – essential)	84.1%
Objectives of citizen involvement (*n* = 22)	
Understand community perspectives/needs	95.5%
Help identify priorities and agenda setting	81.8%
Identify solutions	63.6%
Build public trust/accountability /transparency	63.6%
Raise public awareness/understanding of specific issues	45.5%
Obtain feedback about strategy/policy/programs	45.5%
Increase public support for actions	45.5%
Promote behaviour change	40.9%
Build community capacity	40.9%
Contribute to policy/program design	36.4%
Monitor/evaluate policy/programs	22.7%
Pilot resources or communications	18.2%
Conduct research	9.1%
Purpose of citizen involvement within day-to-day policy practice (*n* = 18)	
Identifying or defining issues to be addressed (prioritising policy issues)	88.9%
Brainstorming potential solutions	77.8%
Disseminating information	44.4%
Collecting data	27.8%
Recruiting other participants	22.2%
Analysing data and/or forming data-based conclusions	16.7%
Identifying questions that need answer from research	11.1%
Designing research methods	5.6%

In day-to-day practice of policy formulation and policymaking, the most frequent reasons to involve the public are ‘identifying or defining issues to be addressed’ or ‘brainstorming potential solutions’.

Policymakers explained these answers in the follow-up interviews: “Such initiatives with citizens … provide us understanding of the issues at hand.” (P7); “In order to make policymaking effective, it is important to connect well with the audience. … We try to collaborate with local stakeholders.” (P7); “To gain insight in what would really meet citizens’ needs. And by giving them the opportunity to provide solutions and ideas, instead of me, the policy officer, doing this. That remains a challenge, right?” (P13); “For public support indeed, but also for doing the right things, for encouraging citizens to take initiatives.” (P4). Citizen involvement is less often employed for (a) the design, monitoring or evaluation of policy programs; (b) for public or community outreach; (c) or for undertaking research. These findings show that citizen involvement mainly ensures that health policy decisions are well-informed and benefit from professional as well as experience knowledge. It reflects a commitment to incorporating diverse perspectives and expertise in the policy decision-making process.

### 3.4 Methods for citizen involvement

**Figure 1:**
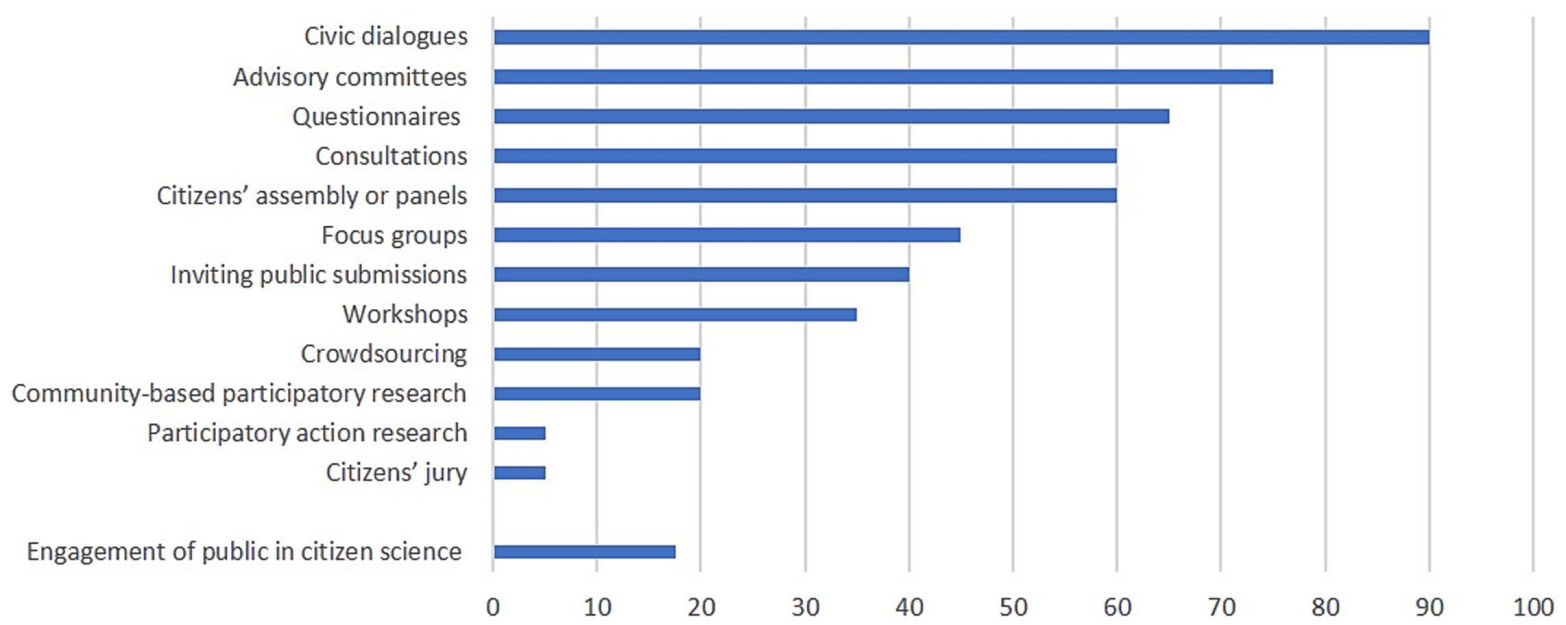
Methods for citizen involvement based on closed-ended questions, as reported by policymakers from the municipalities involved in the study (*n* = 20). *Two policymakers did not answer this question. The response categories ‘Great deal – Quite a lot – Somewhat’ were compared with ‘Not at all – A little’. See Supplementary Appendix 1 for a detailed explanation of the methods for citizen involvement according to the literature.

Civic dialogues, advisory committees, questionnaires, panels or assemblies and consultations are the methods most commonly used for citizen involvement in preventive health policy (Figure 1).

Policymakers elaborated on these methods by saying: “Some of my colleagues are manager of a certain neighbourhood. They work at the town hall, but go on regular outreach to their neighbourhood, to meet with citizens and other local stakeholders (P14).” The participation method can occur either as a one-time event or repeatedly, and it also somewhat determines the number of participants who might be involved. “We have a monthly advisory council with citizens. That’s mandatory, according to the Participation Act.” (P2). “We mainly use questionnaires, that is very traditional, … we are trying to change this as it takes a long time before we receive answers … so now, we also organize meetings with residents, have civic dialogues (P19). “For about two years, we have been using the citizens’ panel which included 200 residents. We approach this panel six times a year, for all kinds of topics concerning welfare policy” (P17).

Small municipalities seem to choose for direct methods compared to larger municipalities. “The advantage of a small municipality is that we are in very close contact with the citizens. Directly as well, you know? Our alderman walks through the neighborhoods and is also approached in the supermarket, as a matter of speaking.” (P5).

Municipal policymakers used these methods for citizen involvement in direct contact with citizens but also indirectly in collaboration with local organizations or stakeholders who are in frequent contact with citizens. They act as representatives for citizens and also function as intermediaries, facilitating citizens’ participation. Additionally, certain municipalities collaborate with external entities for citizen involvement, such as local public health services, universities of applied sciences, or consultancy firms, enhancing the breadth and depth of involvement opportunities. “We occasionally conduct information sessions and engage with network groups, addressing issues like loneliness … In the realm of health, our primary collaboration is through welfare organizations, which, in turn, actively involve citizens.” (P3) “For our municipality, it is convenient to organize citizen involvement with an intermediary. Those intermediaries organize the meetings with residents and collect the data. That makes it easier for us to manage citizens’ expectations … We cannot make promises about future policy during those meetings with citizens.” (P21).

Selecting the appropriate method for citizen involvement requires careful consideration of factors such as the target audience, objectives and resources available. “The method should be attractive for citizens otherwise they will not respond.” (P5). “Regular questionnaires among citizens give us insight in trends … whereas focus groups are more focused on the moment, on certain population group.” (P1). The choice of a method can influence who will participate. Insights into the specific target audience including participations preferences can help to choose the right method. “If the same method of citizen involvement is used, the same kind of people will participate.” (P10) To gain insight in the views of the public on certain topics, municipalities make use of apps, online tools or other creative methods, such as photo voice (P6) or theatre show (P10).

### 3.5 Main challenges of citizen involvement

Allocation sufficient time by policymakers for citizen involvement and ensuring data quality were the main challenges for meaningful citizen involvement within the policymaking process (Table 3). Time constraints may limit the opportunities for extensive participation consultations, feedback gathering, appropriate solutions or collaborative decision-making.

**Table 3:** Challenges for citizen involvement as reported by policymakers from the municipalities involved in the study (*n* = 22), 2023.

Challenges for citizen involvement	Total
Challenges	
Time commitment	70.0%
Ensuring quality of data	60.0%
Resourcing and/or expertise	47.4%
Governance (lack of control over the process)	31.6%
Alignment with organizational priorities	36.8%
Ethics	21.1%
Data ownership and use	21.1%
Scale of projects (e.g., local vs. population wide)	21.1%

“If you really want to engage citizens, you will have to allocate time. For example, we had an intervention in primary schools but we did not have the time for a follow-up meeting with parents.” (P1) “We prefer to involve citizens before the start of a policymaking process, but due to time constraints we cannot manage it always … and when I got them involved, what’s next? … In my organization, I lost control of the process… then it’s going to consume even more of my time.” (P3).

The challenge of ‘ensuring data quality’ was often mentioned with regard to low response rate and selective response. “When we organize a meeting with partners, we notice quite a low number of citizens attending. If the response rates are low, how reliable are the outcomes? To what degree are findings representative for the population?” (P4) “The disadvantaged groups tend to be slightly harder to reach.” (P6). “When I organize a citizens’ meeting, I can predict who will attend, for almost 40 of the 50 participants, those citizens have gained professional-level experience in community consultation processes.” (P1).

Sometimes policymakers struggled to organize citizen involvement, how to reach certain population groups. They sometimes missed the right resources or expertise such as necessary knowledge of cultural aspects which might influence the decision and process of participation: “Some citizens are hard to connect to, for example because of another cultural background or lack of trust in the government.” (P14). “Nor does an unambiguous approach on citizen involvement within the municipality exist … we should have a guideline how we could organize this.” (P16).

Moreover, some organizational challenges included governance issues such as ‘a lack of control over the process’ and ‘alignment with organizational priorities’. “Well, we should do something with the information we gather. That is quite difficult, collecting wishes and needs is not the same as everything is allowed or possible.” (P5). “We have to be patient, sometimes a lot of data is gathered, but we cannot embed all in my organization.” (P7).

Another challenge is the translation of citizens’ perspectives into policy. This would depend on the degree and timing of participation in the different steps of the policy cycle - drafting, implementation or evaluation of policy. Also comments on ‘ethics’ and ‘scale of the projects’ were made: “I have to manage the expectations of citizens. Some participants behave like activists, promoting their own ideas and needs … co-creation is okay, but it must be feasible and effective too” (P2). In our small municipality, we often choose to talk directly with citizens, for example on the marketplace, instead of organizing meetings or surveys.” (P9).

### 3.6 Concerning preventive health policy

Some policymakers found it challenging to apply citizen involvement in the domain of preventive health. “Prevention is complicated; how do we attract citizens to healthy behaviour, … to address their needs … how to involve citizens in that area, that’s a challenge.” (P4). Other policymakers note that poor health is often associated with lower income, poverty, low social economic status, poor literacy, mental health issues or dysfunctional relationships. “How can we reach those citizens? They are very occupied with their circumstances.” (P14). In addition one participant argue: “For a proper understanding of citizens’ contributions during a meeting, it is necessary to learn about the context and background of certain people groups.” (P15).

Policymakers should understand the impact of the social and physical environment on citizens. Some participants mentioned the collaboration with key persons to engage social groups. For instance, key persons can effectively engage the target group by communicating in the language and at (digital) literacy level which resonates with them. Hence, participants recognize that effective communication tailored to the target audience is essential for ensuring understanding and involvement in policy initiatives. (P20).

Moreover, different location and environments of participation are mentioned. One municipality organizes a ‘prevention cafe’ meeting on a regular basis: “Anyone can join and propose a prevention topic … it is often very practical, about a certain initiative. Various stakeholders are involved in the prevention café … it is a platform which produces various citizens’ initiatives.” (P5). Some municipalities collaborate with formal actors in the neighbourhood such as a health care centre.

## 4 Discussion

Noncommunicable diseases are largely driven by preventable risk factors, prompting international and national calls for enhanced efforts to promote population health through tailored, multisectoral preventive policies. Citizen involvement is increasingly recognized as essential in shaping effective, inclusive and legitimate health policies, yet remains complex due to challenges in representativeness, integration into policymaking processes, and resource constraints. This study explores how local policymakers in the Netherlands involve citizens in health promotion, focusing on their motivations, methods, and key challenges. Our study draws on three complementary theoretical frameworks; participatory governance (24), Street-Level Bureaucracy (25) and ‘Normalisation Process Theory’ (26). It takes a public administration perspective within the field of prevention and health promotion, without addressing the broader machinery of policymaking (10, 29). The patterns we observed in our study regarding motivations, methods of citizen involvement, and challenges were consistent with those identified in prior studies. Dutch policymakers predominantly involved citizens in health promotion to better understand community needs, set priorities, and co-develop solutions (9, 13). The most common motives included enhancing the relevance and effectiveness of policies, as well as fostering public trust, accountability and transparency in decision-making processes (7, 9, 10, 22). Citizen involvement in our study typically occurred in the early stages of policymaking — such as identifying issues and brainstorming potential solutions—while it is less frequently applied to program design, monitoring, or evaluation as previously reported (10, 35). The structural integration of citizen input into health policymaking as reported in our study is still limited. To achieve effective policy outcomes, citizen involvement must be carefully planned within the policymaking process and supported by a transparent communication strategy that clearly articulates its objectives, timeline, and anticipated value (10, 16, 35).

Dutch policymakers employed a variety of methods to involve citizens, either directly or via local stakeholders. Some collaborated with regional public health services, university faculties of applied sciences, or consultancy firms. In smaller municipalities, citizen involvement tended to develop more organically, whereas in larger municipalities, it was typically more formalized and structured. This distinction is also observed in other forms of collaborative governance networks (14, 36). Policymakers emphasized the need for better insight in methods of citizen involvement, effective for their target population, policy objectives and available resources. Given the described socioeconomic differences in the acceptability of, and preferences for, health-related policies, it is crucial to systematically include the voices of people in lower socioeconomic positions to ensure that their needs are adequately addressed (37). Our findings point to the need for practical instruments that support policymakers in designing and implementing participatory processes. These may include locally adaptable toolkits, training modules, and guidelines that clarify the purpose, scope, and methods of citizen involvement, and help match them to specific policy stages and target populations. Such support is particularly relevant given the variation in resources and expertise across municipalities (21, 38). Dutch policymakers also faced commonly known challenges in involving citizens in decision-making, including time and resources constraints, concerns about data quality, and limited expertise in participatory methods (10). To address these barriers, municipalities should develop strategies to reach disengaged citizens – for example, by employing a diverse mix of involvement activities and by recruiting citizens via informal leaders, supported by professionals with expertise in citizen involvement (16, 35).

These findings resonate with theoretical insights from the fields of participatory governance and collaborative policymaking. Participatory governance literature emphasizes the shift from government to governance, where decision-making processes increasingly involve non-state actors, including citizens, in a deliberative manner (24) In this light, the limited structural integration of citizen input observed in our study reflects a known tension: while participation is valued in principle, institutional routines, time constraints, and accountability structures often hinder its meaningful implementation (39, 40).

The variation we observed between small and large municipalities can also be interpreted through the lens of collaborative capacity. Smaller municipalities often relied on more informal, relationship-based participation while larger municipalities tended to adopt more formalized structures. This suggests that context-specific capacities, including staff expertise, network density, and administrative flexibility, shape how citizen involvement is operationalized on the ground.

Although our study focuses on the Dutch context, many of the observed patterns—such as reliance on early-stage consultation, difficulties in reaching marginalized groups, and practical barriers to sustained involvement—are consistent with international research on citizen participation in local health policy (41). However, the relatively high degree of decentralisation in the Dutch system, combined with the legal emphasis on local autonomy, may offer municipalities more room to experiment with participatory approaches compared to more centralised governance models in other EU countries.

### 4.1 Strengths and limitations

A strength of the study is that it included municipalities from all regions of the Netherlands, representing a range of population sizes. By covering all regions, the study reduces the risk that the findings are specific to a particular area (e.g., the urban west versus the rural north), thereby increasing the potential generalisability of the results. Additionally, we adapted a citizen involvement questionnaire from a national Australian study (17) that encompassed a broader range of public health professionals, providing a solid foundation for the in-depth exploration of citizen involvement during our interviews.

While the questionnaire was designed to cover a diverse range of engagement methods and underlying rationales, some variation in the interpretation of specific terms may have occurred among respondents in our study, potentially introducing minor noise into the data. To address this, we verified the main findings with policymakers during the interviews, which reduces the risk of misinterpretation and ensures that the researchers’ conclusions align with participants’ perspectives, thereby enhancing the robustness and practical relevance of the results. More importantly, the primary purpose of the questionnaire in our study was not to collect validated or generalisable quantitative data. Rather, it was used as a preparatory tool to help participants reflect on their local practices and challenges regarding citizen involvement.

Our study has some limitations too. First, we interviewed only one policymaker from each municipality regarding citizen involvement in policymaking. Misreporting of citizen involvement activities might occur if the participating policymaker did not have a complete overview. Furthermore, since only one-third of the invited municipalities participated, there is a potential for selection bias. The findings may reflect more positive views on citizen involvement, as participating municipalities are likely those already engaged in such practices to some extent. Although data saturation was reached across a diverse sample, further research is needed to explore whether additional challenges or perspectives exist in municipalities that were not represented.

No responses were received from the provinces of Friesland and Noord-Holland, slightly limiting geographic representation. However, the inclusion of municipalities with similar characteristics from other provinces likely compensates for this, making the findings broadly relevant. In addition, future research would benefit from involving both citizens and policymakers, in order to better understand and enhance citizen participation in health policymaking. This should ideally be explored across a range of municipalities varying in size and context (e.g., rural versus urban settings).

Despite the mentioned limitations, this study highlights the challenges municipalities face in including citizens’ perspectives in health policymaking and identifies what is needed to strengthen citizen involvement.

## 5 Conclusion

Since the late 1980s, the Dutch Government has decentralized health policy mandates to municipalities to foster local citizen involvement in health policy. This study explored policymakers’ current experiences with citizen involvement in preventive health, highlighting the persistent gap between the intent and the practical implementation of citizen engagement in preventive health. It reveals that despite systemic barriers—such as political pressures, institutional inertia, and limited resources—local policymakers increasingly recognize the strategic value of citizen input in shaping responsive health policies. This study contributes to the field by demonstrating that meaningful citizen involvement is not only desirable but also feasible under certain organizational conditions. For policymakers, this implies the need to invest in internal capacity—both in terms of staff expertise and time—and to embed citizen participation structurally into policy processes rather than treating it as an *ad hoc* activity.

From these findings, we recommend: a. Establishing long-term frameworks for citizen engagement that go beyond project-based participation; b. Allocating dedicated resources and training to support participatory processes; c. Creating accountability mechanisms to ensure citizen input influences final policy decisions. Future research should delve deeper into context-specific mechanisms that enable or hinder meaningful engagement, especially in municipalities with varying political and socioeconomic contexts. Additionally, longitudinal studies could track how sustained citizen involvement impacts health outcomes and trust in public institutions over time.

## Data Availability

The raw data supporting the conclusions of this article will be made available by the authors, without undue reservation.
