# Supplementary material for: How do policymakers involve citizens in advancing health? A mixed-method qualitative study in municipalities in the Netherlands
Source: Front Public Health. 2025 Nov 24;13:1708209. doi: 10.3389/fpubh.2025.1708209 (PMC12682865; doi:10.3389/fpubh.2025.1708209)
Supplement: Supplementary file 1 [file Table_1.docx]

APPENDIX 1 Questionnaire Citizen involvement in prevention policy and practice [(15,33)](https://sciwheel.com/work/citation?ids=17023005,18247386&pre=&pre=&suf=&suf=&sa=0,0&dbf=0&dbf=0)

| 1. Does your organisation have a focus on health and wellbeing, for example, health promotion, healthy environments or preventive health? | - Yes |
| --- | --- |
|  | - No |
| 2. Do you have a role that includes policy or program planning, management or decision making? | - Yes |
|  | - No |
| 3. To what extent is public engagement *(deliberate strategies for involving members of the public in research and policy processes)* considered a priority within your organisation? | - Not a priority |
|  | - Low priority |
|  | - Medium priority |
|  | - High priority |
|  | - Essential |
| 4. To what extent does your organisation engage with members of the public in practice? | - Not at all |
|  | - Very little |
|  | - Somewhat |
|  | - Quite a lot |
|  | - A Great deal |
| 5. What are the main objectives of public engagement activities in your organisation?  (*Please select all boxes that apply*) | - To understand community perspectives/needs |
|  | - To help identify priorities and agenda setting |
|  | - To identify solutions |
|  | - To conduct research |
|  | - To raise public awareness/understanding of specific issues |
|  | - To monitor/evaluate policy/programs |
|  | - To contribute to policy/program design |
|  | - To obtain feedback about strategy/policy/programs etc. |
|  | - To increase public support for actions |
|  | - To build public trust/accountability/transparency |
|  | - To pilot resources or communications |
|  | - To promote behaviour change |
|  | - To build community capacity |
|  | - Other (please specify) ______ |
| 6. How frequently does your organisation use the following methods to engage the public? | *1notatall 2verylittle 3somewhat 4quitealot 5agreatdeal* |
| - Questionnaires | 1 not \| 2 \| 3 \| 4 \| 5 a great deal |
| - Focus groups | 1 not \| 2 \| 3 \| 4 \| 5 a great deal |
| - Workshops | 1 not \| 2 \| 3 \| 4 \| 5 a great deal |
| - Civic dialogues | 1 not \| 2 \| 3 \| 4 \| 5 a great deal |
| - Consultations | 1 not \| 2 \| 3 \| 4 \| 5 a great deal |
| - Inviting public submissions | 1 not \| 2 \| 3 \| 4 \| 5 a great deal |
| - Citizens’ assembly or panels | 1 not \| 2 \| 3 \| 4 \| 5 a great deal |
| - Citizens’ juries | 1 not \| 2 \| 3 \| 4 \| 5 a great deal |
| - Community-based participatory research | 1 not \| 2 \| 3 \| 4 \| 5 a great deal |
| - Participatory action research | 1 not \| 2 \| 3 \| 4 \| 5 a great deal |
| - Citizen science | 1 not \| 2 \| 3 \| 4 \| 5 a great deal |
| - Crowdsourcing | 1 not \| 2 \| 3 \| 4 \| 5 a great deal |
| - Advisory committees | 1 not \| 2 \| 3 \| 4 \| 5 a great deal |
| - Other (please specify |  |
| 7. How frequently does your organisation engage members of the public for the following purposes? | *1notatall 2verylittle 3somewhat 4quitealot 5agreatdeal* |
| - Identifying or defining issues to be addressed (prioritising policy issues) | 1 not \| 2 \| 3 \| 4 \| 5 a great deal |
| - Identifying questions that need answer from research | 1 not \| 2 \| 3 \| 4 \| 5 a great deal |
| - Designing research methods | 1 not \| 2 \| 3 \| 4 \| 5 a great deal |
| - Recruiting other participants | 1 not \| 2 \| 3 \| 4 \| 5 a great deal |
| - Collecting data | 1 not \| 2 \| 3 \| 4 \| 5 a great deal |
| - Analysing data and/or forming data-based conclusions | 1 not \| 2 \| 3 \| 4 \| 5 a great deal |
| - Brainstorming potential solutions | 1 not \| 2 \| 3 \| 4 \| 5 a great deal |
| - Disseminating information | 1 not \| 2 \| 3 \| 4 \| 5 a great deal |
| 8. How would you rate the value of public engagement for your work? | 1 Not at all \| 2 slightly \| 3 somewhat \| 4 very \| 5 extremely |
| 9. What do you see as the main value of public engagement in your work? |  |
| Within this survey, we define “citizen science” as a broad approach to public engagement that actively involves members of the public as collaborators in scientific research (e.g. in collecting and analysing data, identifying research questions, brainstorming solutions and advocating for actions). | |
| 10. Does your organisation engage in activities consistent with the definition of citizen science above? If yes, how often does your organisation use these approaches (whether directly, or through third parties)? | - No / Never |
|  | - Rarely |
|  | - Sometimes |
|  | - Often |
|  | - Always |
| 11. What are the main objectives of citizen science activities in your organisation?  *Please select all boxes that apply* | - To understand community perspectives and/or needs |
|  | To help identify priorities and agenda setting |
|  | - To identify solutions |
|  | - To conduct research |
|  | - To raise public awareness /understanding of issues |
|  | - To monitor or evaluate policy or programs |
|  | - To contribute to policy or program design |
|  | - To obtain feedback about strategy, policy, programs etc. |
|  | - To increase public support for actions |
|  | - To build public trust, accountability or transparency |
|  | - To pilot resources or communications |
|  | - To promote behaviour change |
|  | - To build community capacity |
|  | - Other ……………… |
| 12. To what extent do you think citizen science approaches might be a useful way to… | 1 Not at all 2 Slightly 3 Somewhat 4 Very 5 Extremely |
| - Increase public understanding about issues | 1 not \| 2 \| 3 \| 4 \| 5 extremely |
| - Generate useful data | 1 not \| 2 \| 3 \| 4 \| 5 extremely |
| - Increase public support for policy or programs | 1 not \| 2 \| 3 \| 4 \| 5 extremely |
| - Build public trust, accountability | 1 not \| 2 \| 3 \| 4 \| 5 extremely |
| - Build community capacity for action | 1 not \| 2 \| 3 \| 4 \| 5 extremely |
| - Improve the relevance of research | 1 not \| 2 \| 3 \| 4 \| 5 extremely |
| - Improve policymaking | 1 not \| 2 \| 3 \| 4 \| 5 extremely |
| - Improve evidence-based practice | 1 not \| 2 \| 3 \| 4 \| 5 extremely |
| 13. What do you see as the main challenges of citizen science approaches and public engagement)? (*Please select all boxes that apply*) | - Ensuring quality of data |
|  | - Ethics |
|  | - Resourcing and/or expertise |
|  | - Governance e.g. lack of control over the process |
|  | - Alignment with organisational priorities |
|  | - Time commitment |
|  | - Data ownership and use |
|  | - Scale of projects (e.g. local vs. population-wide) |
|  | - Other …. |
| A few questions about you and the nature of your work. |  |
| - What is your age | …. year |
| - How would you describe your gender? | Male Female Other |
| - In which organisation do you work? |  |
| - What is your workplace (name city/area)? |  |
| - At what level of policy or practice do you work? | Local, regional or national |
| - What is your occupation? | - Policy officer/analyst |
|  | - Senior policy officer |
|  | - Program manager |
|  | - Research officer/manager |
|  | - Policy/Program Director |
|  | - Senior manager/Executive |
|  | - Other |

*Short description of the method, from the literature. This was not included in the questionnaire for participants*

| - Civic dialogue is a structured format of public engagement that fosters understanding among diverse groups. It can build broad-based consensus and commitment around complex or controversial issues. Civic dialogue can be applied in various contexts to support productive communication. |
| --- |
| - Workshops involve in-depth, informed discussions on complex or controversial issues. They are used to gather social intelligence to inform policy, anticipate regulations, exchange opinions, or raise awareness. Deliberative workshops are also used to develop research agendas and objectives that better reflect public perspectives. These workshops evolved from focus groups and related methods as a more thorough and deliberative approach. - Future Workshops are a method for visioning and planning the future of a specific geographic area. These workshops help local stakeholders define goals and identify problems collaboratively. |
| - A focus group is a qualitative research method used to explore people’s preferences or to evaluate strategies and concepts. Originally developed for market research, participants are selected based on shared characteristics relevant to the research topic. Groups typically consist of 8–10 individuals. Focus groups are often used to generate or assess hypotheses and ideas. |
| - A citizens’ assembly is a group of citizens brought together to deliberate on issues of local, regional, or national importance. Its goal is to ensure a representative selection of the public who can learn about a topic, consider options, and make recommendations—independently of political or policymaker influence. |
| - A citizens’ jury is convened to advise a governmental body on a policy-relevant issue. Within the jury, the perspectives of citizens—who are all stakeholders—are explored, and a shared recommendation is developed. Citizens’ juries are particularly suited to issues that have multiple potential solutions, allowing participants to evaluate and deliberate on these policy options. |
| - Consultations are processes through which governments actively seek the opinions, perspectives, and input of citizens regarding specific issues, policies, or decisions. These processes involve listening to the public and, potentially, acting on their feedback. |
| - In community-based participatory research, the community is involved in all stages of the research process—from defining the research questions and conducting the study to interpreting and communicating the results. The research is aimed at understanding and improving specific local situations. When combined with actions to implement the findings, this method leads to a cycle of participatory action research. |
| - Participatory action research integrates research into society by democratizing knowledge production and grounding it in real community needs. It is widely used in areas such as education, public health, labour, and development cooperation. Unlike citizen science, this method emphasizes both public engagement and transformative action by involving citizens in analysing and addressing their own everyday issues and environmental challenges. It is a communicative process that recognizes multiple valid forms of knowledge—including both scientific and citizen-based knowledge. |
| - Citizen science involves the participation of non-professionals in scientific research—either by asking questions, collecting data, or analyzing findings. Citizens are actively involved in the research process, not just as subjects but as contributors. Today, many citizen science projects are organized and supervised by professional scientists and often involve large-scale data collection (e.g., environmental monitoring or biodiversity surveys) or repetitive tasks that do not require specialized expertise. |
| - Crowdsourcing is the practice of engaging a broad audience—often through online platforms—to contribute ideas, knowledge, or solutions to a public issue, policy challenge, or project. |
| - This involves asking individuals, community groups, organizations, or stakeholders to provide written feedback—such as comments, ideas, concerns, or recommendations—on a specific topic within a defined timeframe. |
| - A questionnaire is a research instrument consisting of a series of open-ended or closed-ended questions used to systematically collect information from citizens. |
| - An advisory committee consists of citizens selected for their knowledge, expertise, and diverse perspectives. These committees provide informed advice to policymakers and other decision-makers. |
