# Supplementary material for: How do policymakers involve citizens in advancing health? A mixed-method qualitative study in municipalities in the Netherlands
Source: Front Public Health. 2025 Nov 24;13:1708209. doi: 10.3389/fpubh.2025.1708209 (PMC12682865; doi:10.3389/fpubh.2025.1708209)
Supplement: Supplementary file 2 [file Table_2.docx]

APPENDIX 2 TABLE Characteristics of invited and participating municipalities, 2023

|  |  | Total |  | Small ^*^ | Medium ^*^ | Large ^*^ | Small ^*^ | Medium ^*^ | Large ^*^ |
| --- | --- | --- | --- | --- | --- | --- | --- | --- | --- |
|  |  |  |  |  |  |  |  |  |  |
|  |  | n | % | n |  |  | % |  |  |
| Total |  | 342 |  | 243 | 67 | 32 | 71.1 | 19.6 | 9.4 |
| Invited |  | 65 | 100.0 | 52 | 10 | 3 | 80.0 | 15.4 | 4.6 |
| Participating |  | 22 | 33.8 | 16 | 5 | 1 | 72.7 | 22.7 | 4.5 |
|  |  |  |  |  |  |  |  |  |  |
| Provinces |  |  |  |  |  |  |  |  |  |
| 1. Friesland | Total | 18 |  | 13 | 4 | 1 | 72.2 | 22.2 | 5.6 |
|  | Invited | 3 | 100.0 | 3 | 0 | 0 | 100.0 | - | - |
|  | Participating | 0 | 0.0 | 0 | 0 | 0 |  |  |  |
| 1. Groningen | Total | 10 |  | 7 | 2 | 1 | 70.0 | 20.0 | 10.0 |
|  | Invited | 2 | 100.0 | 2 | 0 | 0 | 100.0 | - | - |
|  | Participating | 1 | 50.0 | 1 | 0 | 0 | 100.0 | - | - |
| 1. Drenthe | Total | 12 |  | 9 | 2 | 1 | 75.0 | 16.7 | 8.3 |
|  | Invited | 2 | 100.0 | 1 | 0 | 1 | 50.0 | - | 50.0 |
|  | Participating | 1 | 50.0 | 0 | 0 | 1 | - | - | 100.0 |
| 1. Overijssel | Total | 25 |  | 18 | 4 | 3 | 72.0 | 16.0 | 12.0 |
|  | Invited | 5 | 100.0 | 3 | 1 | 1 | 60.0 | 20.0 | 20.0 |
|  | Participating | 2 | 40.0 | 1 | 1 | 0 | 50.0 | 50.0 | - |
| 1. Gelderland | Total | 51 |  | 44 | 3 | 4 | 86.3 | 5.9 | 7.8 |
|  | Invited | 10 | 100.0 | 9 | 1 | 0 | 90.0 | 10.0 | - |
|  | Participating | 5 | 50.0 | 5 | 0 | 0 | 100.0 | - | - |
| 1. Flevoland | Total | 6 |  | 4 | 1 | 1 | 66.7 | 16.7 | 16.7 |
|  | Invited | 1 | 100.0 | 1 | 0 | 0 | 100.0 | - | - |
|  | Participating | 1 | 100.0 | 1 | 0 | 0 | 100.0 | - | - |
| 1. Utrecht | Total | 26 |  | 14 | 10 | 2 | 53.8 | 38.5 | 7.7 |
|  | Invited | 5 | 100.0 | 4 | 1 | 0 | 80.0 | 20.0 | - |
|  | Participating | 3 | 60.0 | 2 | 1 | 0 | 66.7 | 33.3 | - |
| 1. Noord-Holland | Total | 44 |  | 30 | 9 | 5 | 68.2 | 20.5 | 11.4 |
|  | Invited | 8 | 100.0 | 6 | 2 | 0 | 75.0 | 25.0 | - |
|  | Participating | 0 | 0.0 | 0 | 0 | 0 |  |  |  |
| 1. Zuid-Holland | Total | 50 |  | 27 | 15 | 8 | 54.0 | 30.0 | 16.0 |
|  | Invited | 10 | 100.0 | 8 | 2 | 0 | 80.0 | 20.0 | - |
|  | Participating | 2 | 20.0 | 1 | 1 | 0 | 50.0 | 50.0 | - |
| 1. Zeeland | Total | 13 |  | 11 | 2 | 0 | 84.6 | 15.4 | - |
|  | Invited | 2 | 100.0 | 1 | 1 | 0 | 50.0 | 50.0 | - |
|  | Participating | 2 | 100.0 | 1 | 1 | 0 | 50.0 | 50.0 | - |
| 1. Noord Brabant | Total | 56 |  | 42 | 10 | 4 | 75.0 | 17.9 | 7.1 |
|  | Invited | 11 | 100.0 | 9 | 2 | 0 | 81.8 | 18.2 | - |
|  | Participating | 3 | 27.3 | 3 | 0 | 0 | 100.0 | - | - |
| 1. Limburg | Total | 31 |  | 24 | 5 | 2 | 77.4 | 16.1 | 6.5 |
|  | Invited | 6 | 100.0 | 5 | 1 | 0 | 83.3 | 16.7 | - |
|  | Participating | 2 | 33.3 | 1 | 1 | 0 | 50.0 | 50.0 | - |
|  |  |  |  |  |  |  |  |  |  |

^* Small < 50.000 inhabitants, medium 50.000-100.000, large >= 100 thousands [17. CBS, 2023].^
